# A technique for esophagojejunostomy following robot-assisted gastrectomy: a liner stapler and barbed suture device-based technique: a case series

**DOI:** 10.1097/MS9.0000000000000407

**Published:** 2023-04-11

**Authors:** Hironori Ohdaira, Teppei Kamada, Junji Takahashi, Keigo Nakashima, Yuichi Nakaseko, Takayuki Ishigaki, Norihiko Suzuki, Masashi Yoshida, Eigoro Yamanouchi, Yutaka Suzuki

**Affiliations:** Departments of a Surgery; b Radiology, International University of Health and Welfare Hospital, Nasushiobara, Tochigi Prefecture, Japan

**Keywords:** anastomosis, esophagojejunostomy, robot-assisted gastrectomy

## Abstract

Intracorporeal esophagojejunostomy after total or proximal robot-assisted gastrectomy is technically more demanding than gastroduodenostomy and gastrojejunostomy for distal gastrectomy, as well as laparoscopic surgery. We have established a safe and simple esophagojejunostomy procedure using a liner stapler attached to the Da Vinci Surgical System and a barbed suture device.

**Patients and methods:**

For esophagojejunostomy after total gastrectomy or proximal gastrectomy with double-tract reconstruction, we choose the “overlap method,” in which entry holes were made at the left of the esophageal stump and at 5 cm of the anal side in antimesentric area of the jejunum, followed by anastomosis on the left of the esophagus using SureForm (blue 45 mm) and hand-sewing closure of the common entry hole with V-Loc. We analyzed the short-term surgical outcomes of all patients.

**Results:**

23 patients underwent this reconstruction technique. None of the patients required any further open surgeries. The mean time to perform anastomosis was 24.7±2.8 min. The postoperative course was uneventful in 22 patients; a single patient developed minor anastomotic leakage (Clavien–Dindo grade 3), which was treated with conservative therapy employing a drainage tube.

**Conclusion:**

Our esophagojejunostomy method following robot-assisted gastrectomy is simple and feasible, with acceptable short-term outcomes, and could represent the procedure of choice for esophagojejunostomy.

## Introduction

Highlights

- Esophagojejunostomy with overlap method for robot-assisted gastrectomy.
- Using a liner stapler attached to the surgical robot with barbed suture closure.
- There were no conversions to open surgery.
- The mean time to perform anastomosis was 24.7±2.8 min.

In Japan, the use of robot-assisted gastrectomy for gastric malignancy using the Da Vinci Surgical System (DVSS) (Intuitive Surgical Inc.) has recently increased, and its indications have expanded to proximal and total gastrectomy^1^. However, intracorporeal esophagojejunostomy after total or proximal, robot-assisted gastrectomy is technically more complex than gastroduodenostomy and gastrojejunostomy in distal gastrectomy and laparoscopic surgery^2^.

We have established a safe and simple esophagojejunostomy following robot-assisted gastrectomy using a liner stapler attached to the DVSS Xi and a barbed suture device.

## Patients and methods

Table 1 shows the patient characteristics of 23 patients that underwent this procedure. We analyzed the short-term surgical outcomes of all patients. Five patients had a Siewert type II adenocarcinoma of the esophagogastric junction and lower mediastinal dissection. Double-tract reconstruction was performed after proximal gastrectomy. The other 18 patients had adenocarcinoma located in upper–middle body of the stomach.

**Table 1:** Patients characteristic and results

Age (years)	70.6±7.5	
Sex (male:female)	15:8	
BMI (kg/m^2^)	22.8±3.9	
Extent of resection	TG: 20	PG: 3
Extent of lymph node dissection	D1+: 4	D2: 19
Level of anastomotic site	Abdomen: 18	Lower mediastinum: 5
Closure of the entry holes	Single layer: 15	Two layer: 8
Operation time (min)	411.5±42.6	
Time to perform anastomosis (min)	24.7±2.8	
Complication-related anastomosis	Leakage	1
	Stenosis	0

The data were expressed as mean±SD or number.

PG, proximal gastrectomy (double-tract reconstruction); TG, total gastrectomy.

For esophagojejunostomy, we chose and modified the ‘overlap method’^3^–^5^. In this method, entry holes are made at the left of the esophageal stump and at 5 cm of the anal side on the antimesenteric area of the jejunum, followed by anastomosis on the left side of the esophagus, employing a 45-mm endoscopic liner stapler and hand-sewing closure of the common entry hole. A cartridge fork was inserted into the jejunum, and an anvil fork was inserted into the esophagus, guided by a nasogastric tube^2^.

For the linear stapler dedicated to the DDVS Xi, we used a 45 mm blue SureForm (Intutive Surgical Inc.) cartridge. We closed the entry hole using an absorbable barbed suture device (V-Loc; Covidien).

**Figure 1:**
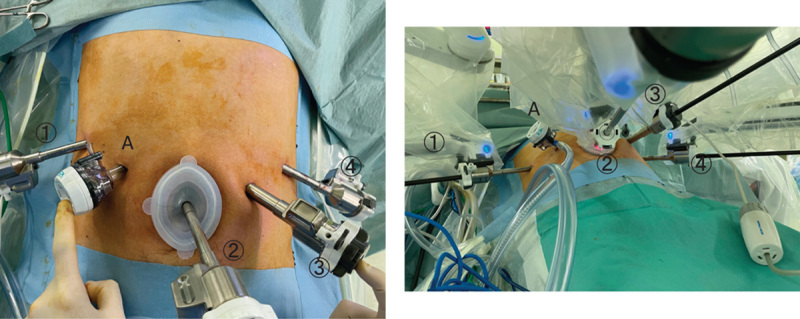
Placement of the cannula. ①: First arm for Fenestrated or Maryland bipolar forceps. ②: Second arm for camera. ③: Third arm for SureForm, monopolar curved scissors, and other energy devices. ④: Fourth arm for Cadiar forceps. A, Assistant port.

Figure 1 shows our cannula placement. Intuitive cannulas of 8 mm were inserted in the right and left lateral hypochondriac regions (① and ④). For the camera scope, 8 mm intuitive cannula were inserted around the umbilicus (②). Trocars of 12 mm were added for a patient-side assistant (A). For insertion of the Sureform, 13 mm intuitive cannula was placed between 8 mm intuitive cannula (④) and the camera cannula (②) along the left mid-clavicular line.

After insertion of the instrument for the SureForm through a 13 mm intuitive cannula, we inserted the cartridge fork of the linear stapler into the opening made in the jejunum and brought up the jejunal limb at the left side of the esophagus.

**Figure 2:**
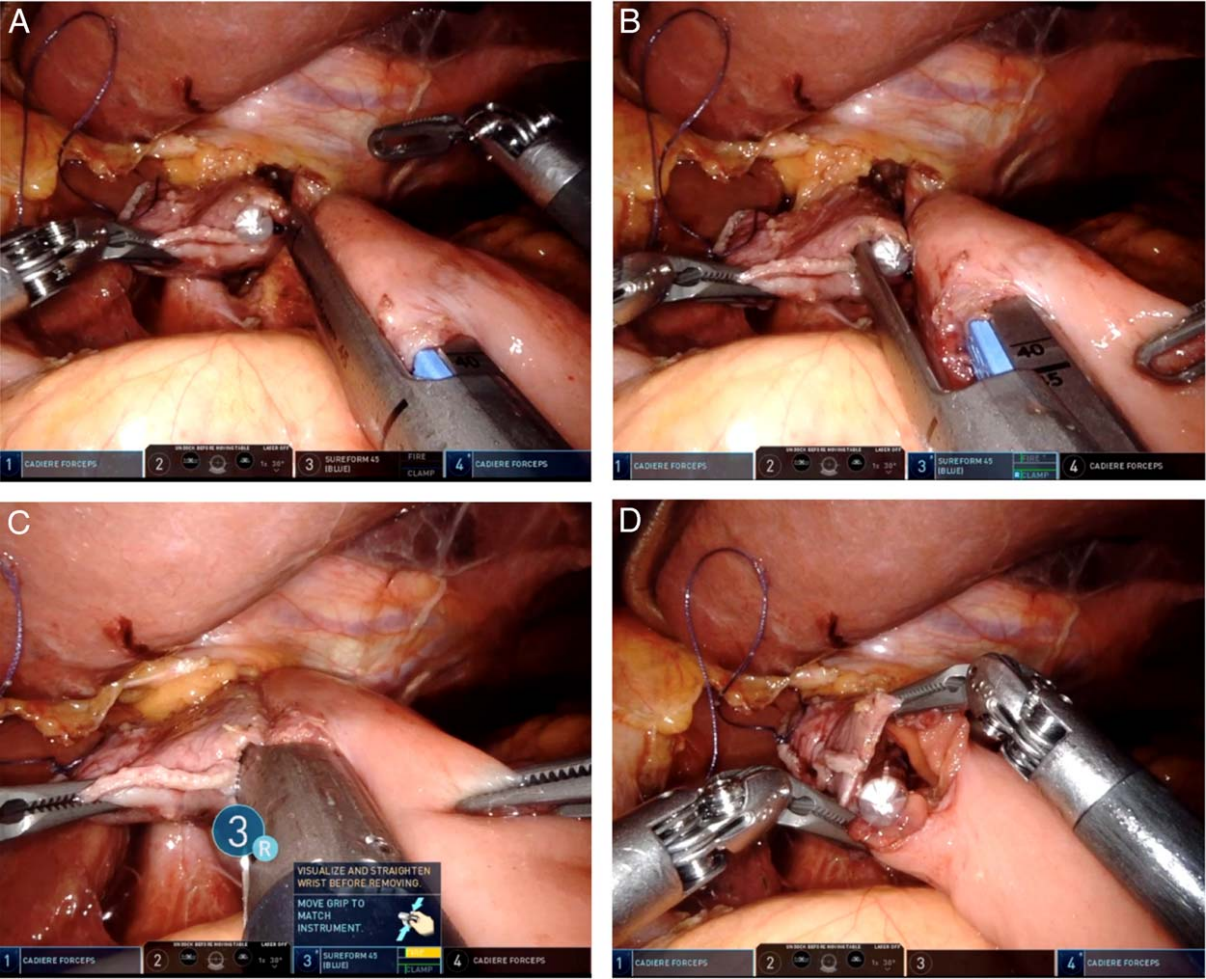
An anastomotic staple line was created between esophageal stump and the jejunal limb. (A) A cartridge fork was inserted into the jejunum. (B) An anvil fork was inserted into the esophagus guided by the nasogastric tube. (C) SureForm was closed and fired between the esophagus and jejunum. (D) An entry hole was created.

We inserted the anvil fork of the linear stapler into the opening of the esophageal stump. After each fork was completely inserted into the lumen, the two limbs were joined together to form a side-to-side esophagojejunal anastomosis. The firing of the stapler converted the two openings into a single-entry hole to create an esophagojejunostomy (Fig. 2).

**Figure 3:**
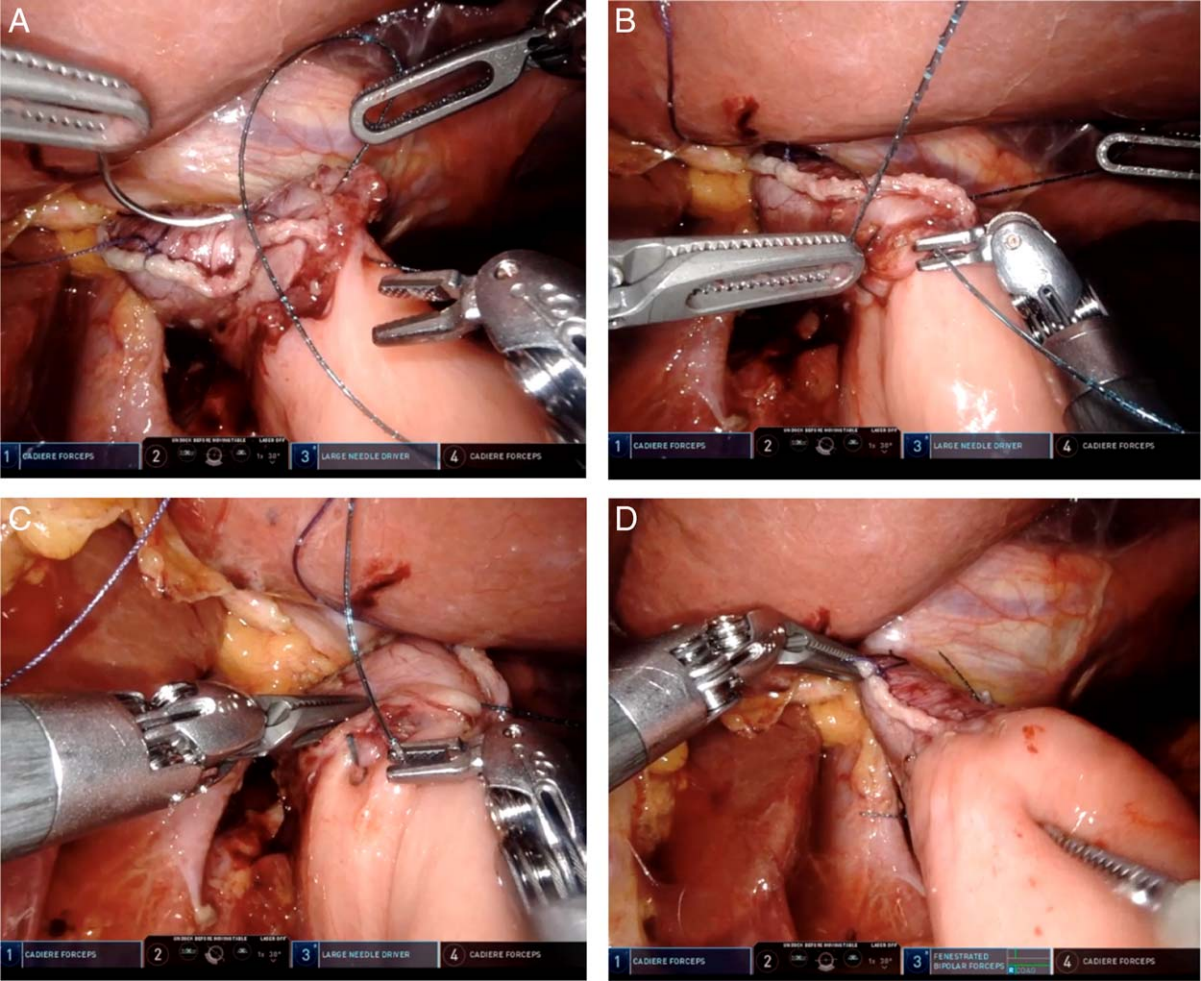
The common entry hole was closed bidirectionally using V-Loc (showing an example for a two-layer close^7^). (A) The first full-thickness inner layer closure starts from the left corner of the staple line between the esophagus and jejunum. (B) The second full-thickness inner layer closure starts from the right corner of the staple line. (C) The second seromuscular layer closure using the first suture directs towards the right corner of anastomosis. (D) Entry hole closure was completed.

The entry hole of the stapler was closed using a continuous hand-sewn suture with V-Loc. Specifically, we employed a single-layer suture^6^ with our modification (Video 1, Supplemental Digital Content 1, http://links.lww.com/MS9/A58) or a two-layer suture^7^ (Video 2, Supplemental Digital Content 2, http://links.lww.com/MS9/A59) (Fig. 3).

This work has been reported in line with the PROCESS criteria^8^ (Supplemental Digital Content 3, http://links.lww.com/MS9/A60).

[Registration of research studies: UMIN (University Hospital Medical Information Network), UMIN000032895, https://center6.umin.ac.jp/cgi-open-bin/icdr_e/ctr_view.cgi?recptno=R000037504].

## Results

The results are presented in Table 1. None of the patients required any further open conventional laparoscopic surgeries. The mean time to perform anastomosis was 24.7±2.8 min, although the total operation time was 411.5±42.6 min. The postoperative course was uneventful for 22 patients. A single patient with the single-layer closure in the abdominal cavity developed minor anastomotic leakage (Clavien–Dindo grade 3), which was treated with conservative therapy using a drainage tube placed during gastrectomy.

## Discussion

Methods for esophagojejunostomy after laparoscopic gastrectomy have been established. In Japan, functional end-to-end anastomosis or the overlap method are mainly used in linear staplers^9^.

When we introduced robot-assisted surgery for gastric cancer, we recognized the difficulty in reconstruction which we did not experience in conventional laparoscopic gastrectomy.

First, the insertion of a stapler into the esophagus and closure of the entry hole were more complicated than in laparoscopic gastrectomy, especially in functional end-to-end anastomosis. We believe that this is due to the cannula placement and the surgical arm of the patient cart. Cannulas of robots are placed more laterally than in conventional laparoscopic surgeries, to avoid surgical arm conflict. Narrow spaces surrounded by four surgical arms interfere with the assistants’ handling of the staplers. Kitagami *et al.* 10 reported that the robotic arms interfere with the surgical field, hence the assistant use the stapler through the space between them in delta anastomosis for robot-assisted distal gastrectomy.

To improve this situation, when we upgraded the surgical robot from DVSS Si to Xi, we introduced a linear stapler attached to the DVSS Xi, SureForm. This stapling device, similar to other robotic instruments, has good access to the target site owing to its articulating function. It can easily pass over the pancreas and the transverse colon and lift the jejunum into the esophagus without assistance.

Another difficulty was encountered while tying the knot of the suture. The surgical robot made needle handling and knot tying easier than laparoscopic surgery. However, considering that knot tying requires familiarity with the lateral movements of the instrument, it could create surgical arm conflict.

We believe that barbed suture devices can overcome this problem. Since the introduction of DDVS, we have used the barbed suture device V-Loc for entry hole closure. Some studies have reported that barbed sutures improve the efficiency and the safety of intracorporeal reconstruction of the digestive tract and reduce its cost by minimizing the time required for suturing to close the entry hole^11^,^12^.

We believe that the results obtained using our method are acceptable. The mean time to anastomosis was 24.7 min. Morimoto et al. reported that the mean time to perform anastomosis was 36 min in conventional laparoscopic esophagojejunostomy^4^. Anastomotic stenosis was not observed, although one minor leakage was treated nonoperatively. Sun *et al.* 13 reported an anastomosis leakage rate of 2.1%.

Our study had two limitations. The sample size of patients was small, and long-term outcomes were not available. Therefore, further studies are required. This study used two different closure methods for the barbed sutures. Based on our results, we believe that both methods are acceptable, as long as all layers of sutures are secured, and the choice could be the surgeon’s preference.

In conclusion, our esophagojejunostomy method following robot-assisted gastrectomy is simple and feasible with acceptable short-term outcomes. This could represent the procedure of choice for esophagojejunostomies.

## Ethical approval

The study protocol was approved by the Ethics Committee for Biomedical. Research of the International University of Health and Welfare Hospital (21-B-22), and informed consent was obtained from all patients.

## Consent

Written consent was obtained by the patients.

## Sources of funding

None to declare.

## Author contribution

All authors contributed equally to the manuscript.

## Conflicts of interest disclosure

The authors declare that they have no financial conflict of interest with regard to the content of this report.

## Research registration unique identifying number (UIN)

1. Name of the registry: UMIN (University hospital Medical Information Network)
2. Unique Identifying number or registration ID: UMIN000032895
3. Hyperlink: https://center6.umin.ac.jp/cgi-open-bin/icdr_e/ctr_view.cgi?recptno=R000037504

## Guarantor

Hironori Ohdairar.

## Provenance and peer review

Not commissioned, externally peer reviewed.

## Supplementary Material
